# Laparoscopic Ovum Pick-Up Followed by In Vitro Embryo Production and Transfer in Assisted Breeding Programs for Ruminants

**DOI:** 10.3390/ani11010216

**Published:** 2021-01-17

**Authors:** Hernan Baldassarre

**Affiliations:** Department of Animal Science, McGill University, Montreal, QC H3A 0G4, Canada; hernan.baldassarre@mcgill.ca

**Keywords:** laparoscopy, in vitro embryo, hormonal stimulation, sheep, goat, cattle, buffalo, prepubertal, accelerated genetic gain

## Abstract

**Simple Summary:**

In vitro embryo production from oocytes collected by laparoscopy has the potential of producing more offspring from genetically superior females of ruminant species (e.g., sheep, goats, cervids) or ages (e.g., prepubertal cattle and buffalo) that are too small to be eligible for oocyte collection by the transvaginal ultrasound-guided method used in cows. This article reviews the multiple applications of the technology, how it is done, the pros and cons, the limitations to widespread use, and the envisioned improvements that are expected in the years to come. In small ruminants, where conventional embryo recovery is most commonly done by surgery, the technology offers a less invasive approach, i.e., more animal welfare-friendly and with minimum risks of surgical sequels. Thereby, it enables repeating the procedure in the same animals exponentially more times, resulting in the potential for an increased number of offspring born from elite donors. Furthermore, the emerging most attractive application is for the in vitro production of embryos from prepubertal animals at very young ages, which allows having progeny born from genetically superior donors before they reach the age and weight to be bred for the first time.

**Abstract:**

The potential of laparoscopic ovum pick-up (LOPU) followed by in vitro embryo production (IVEP) as a tool for accelerated genetic programs in ruminants is reviewed in this article. In sheep and goats, the LOPU-IVEP platform offers the possibility of producing more offspring from elite females, as the procedure is minimally invasive and can be repeated more times and more frequently in the same animals compared with conventional surgical embryo recovery. On average, ~10 and ~14 viable oocytes are recovered by LOPU from sheep and goats, respectively, which results in 3–5 transferable embryos and >50% pregnancy rate after transfer. LOPU-IVEP has also been applied to prepubertal ruminants of 2–6 months of age, including bovine and buffalo calves. In dairy cattle, the technology has gained momentum in the past few years stemming from the development of genetic marker selection that has allowed predicting the production phenotype of dairy females from shortly after birth. In Holstein calves, we obtained an average of ~22 viable oocytes and ~20% transferable blastocyst rate, followed by >50% pregnancy rate after transfer, declaring the platform ready for commercial application. The present and future of this technology are discussed with a focus on improvements and research needed.

## 1. Introduction

Over the past few decades, laparoscopic ovum pick-up (LOPU) has become the golden standard for the recovery of oocytes from live animals that are too small to be eligible for ultrasound-guided transvaginal ovum pick-up [1,2,3,4,5]. The concept was first described by Snyder and Dukelov in 1974, who recovered 6 oocytes from 21 follicles aspirated from an ewe under laparoscopic observation [6]. However, the technology was not fully developed until the early 1990s, stimulated by the development of in vitro embryo production (IVEP) technologies applied to ruminant species [7,8]. The initial target was the collection of oocytes from farm animal species in which ultrasound-guided ovum pick-up (OPU) was either too difficult or not possible, mainly owing to animal size issues. In that regard, LOPU quickly became the method of choice for sourcing immature oocytes for in vitro embryo production in sheep [1,9,10] and goats [3,11,12]. Furthermore, LOPU has also been identified as the method of choice for sourcing oocytes from deer and other wild ruminants in conservation programs [13,14]. Finally, oocyte collection by LOPU has also been used for accelerated genetic gain in prepubertal dairy cattle and buffalo [2,5,15].

Before moving onto the technical details of the technology, it is important to establish “why” LOPU-IVEP is considered an important breeding tool, which will bring up current and envisioned applications. The main interest is accelerated genetic gain through exponential reproduction of females of outstanding productivity and genetic value. Like with multiple ovulation and embryo transfer (MOET), the main goal is the production of more progeny from females that have been demonstrated to be highly superior compared with the herd average. In small ruminant species, LOPU-IVEP has the potential for producing more offspring from valuable females than MOET, because it can be repeated more often and more times in the same animals given that it is a less invasive technology, compared with the surgical methods most commonly used for embryo recovery in these species. Moreover, in small ruminants, MOET has shown to be extremely variable in results, with poor superovulatory response, lack of fertilization, and premature luteolysis identified as the main sources of failure [16,17,18]. In that sense, LOPU-IVEP can be a very helpful alternative for females that have repeatedly failed to produce embryos by MOET for the reasons above, all three of which can be avoided by LOPU-IVEP. Another application of interest is the reproductive rescue of females that are too aged to conceive and/or carry a pregnancy to term, but their ovaries are still active with follicular waves and production of competent oocytes for recovery by LOPU [19]. In yet another application of the technology, LOPU-sourced oocytes have been successfully used as recipient cytoplasts for cloning programs by somatic cell nuclear transfer, as well as for the production of zygotes for microinjection in programs aimed at the production of transgenic and genome-edited animals [20,21,22,23,24,25].

LOPU-IVEP has also been shown to be an excellent technology for accelerated genetic gain through shortening of the generational interval, because it allows the production of progeny from females at prepubertal ages, typically of 2–6 months of age. This application of the technology has been sometimes referred to as juvenile in vitro embryo production (JIVET), and essentially exploits the fact that, although prepubertal females are incapable of ovulation, waves of follicular growth occur and the recruited follicles can be stimulated with exogenous gonadotropins to produce competent oocytes for aspiration, followed by in vitro embryo production. In the case of small ruminants, LOPU-IVEP has been applied to the production of embryos and live births from lambs [26,27] and goat kids [28]; however, this technology has not been widely used for a number of reasons, which will be discussed herein, and the lack of genetic marker technology. The potential of applying LOPU-IVEP technology to the production of “calves from calves” was envisioned in the early 1990s, with multiple publications showing high oocyte yields from bovine calves of 2–6 months of age stimulated with gonadotropins and subjected to LOPU. Many studies reported oocyte yields substantially higher than those of adult cows [4,9,29,30]. However, this initial interest in developing the technology died shortly for two main reasons. First, it was virtually impossible to accurately predict the production phenotype of an animal at 2 months of age. Second, calf oocytes showed very poor developmental competence compared with oocytes from adult cows [31,32]. As a result, efforts to further develop this technology were practically abandoned for two decades. More recently, the interest in developing the technology to a commercial level has resurged. First, with the advent of genomic marker technology, it is now possible to predict the production phenotype of dairy cattle from the moment they are born [33,34]. Consequently, the owners of those valuable animals do not want to wait until those females are old/big enough for ultrasound-guided OPU, and LOPU-IVEP is the method of choice for making pregnancies and offspring from those females at the fastest possible speed. Second, IVEP technologies have improved dramatically in the last three decades, including the possibility of freezing IVEP-blastocysts following direct transfer protocols, and obtaining pregnancy results comparable to those of in vivo produced embryos [35]. Combined, especially in the highly competitive market of dairy genetics, the possibility of producing high quality embryos from elite females as early as 2 months of age has the potential of becoming a breeding target. Genetic gain is maximized with this technology by combining genetic marker selection (highest selection differential) with extremely early breeding of selected animals (shortest generation interval). In addition, from a commercial standpoint, getting to the marketplace faster with new genetics is very attractive, especially for semen companies.

## 2. Hormonal Stimulation

The first step is to hormonally stimulate the females in preparation for LOPU to maximize the number and quality of oocytes collected per donor. Studies have shown that the developmental competence of oocytes increases with follicular size in sheep [36], goats [37,38], heifers and cows [39,40,41], and buffalo [42]. This is consistent with the knowledge that oocyte acquisition of developmental competence requires the accumulation of critical molecules during follicular development, from the dormant primordial to the pre-ovulatory stage. Some molecules are biosynthesized by the oocyte itself and some are provided by the cumulus granulosa cells through transzonal projections and gap junctions. Some molecules are necessary for oocyte growth, some are involved in the cross-talk with cumulus cells, some are needed later for oocyte maturation and fertilization, and some are needed even later to support embryo development until embryonic genome activation [43,44,45]. Hence, it is extremely important to provide a proper exogenous gonadotropin regime that will support follicular development and oocyte acquisition of developmental competence. This is of utmost importance in prepubertal donors given the fact that their hypothalamus–pituitary–ovary axis is not yet fully functional.

### 2.1. Small Ruminants

Estrus synchronization is conducted by means of progesterone or progestogen-containing intravaginal devices (e.g., CIDR; sponges) that are applied for 7–11 days in combination with a luteolytic dose of prostaglandin 48 h prior to LOPU. Different treatments have been proposed for stimulation of follicular growth prior to LOPU in sheep and goats. In the “Multiple FSH” regime, 80–100 mg of FSH (Folltropin^®^, Vetoquinol, Lavaltrie, Canada) is administered at 12 h intervals, starting 36–48 h prior to LOPU. An alternative so-called “Oneshot” protocol combines 80 mg of FSH with 300 IU of eCG administered simultaneously 36–48 h prior to LOPU. As previously reported work has shown no significant differences between these two treatments [46], the Oneshot treatment is considered more convenient owing to its simplicity. More recently, we looked at an alternative “Oneshot-FSH” protocol without co-injecting eCG. The designed treatment used 10 mL of a 0.5% hyaluronic acid solution (MAP-5^®^, Vetoquinol, Lavaltrie, QC, Canada) for recomposing the lyophilized FSH inside the Folltropin^®^ vial. The hyaluronate acts as a slow releasing factor, making FSH move into circulation in a slow manner, and we have hypothesized that this would allow for similar ovarian stimulation results than with the Oneshot protocol, without the need for using eCG, which is known to be immunogenic [47] and is becoming less acceptable for use owing to animal welfare concerns. The results proved the working hypothesis to be correct as the average number of follicles aspirated (goats: 17.8 vs. 17.9; sheep: 12.6 vs. 12.4) and oocytes recovered (goats: 13.7 vs. 14.0; sheep: 10.9 vs. 10.8) were not different between the control and FSH-MAP5 groups [48]. Similar protocols have been successfully used for the hormonal stimulation of prepubertal sheep [27,49,50] and goats [3,28], but without the need for estrus synchronization, as they are not cycling.

### 2.2. Prepubertal Cattle and Buffalo

Early studies clearly established that antral follicles from prepubertal cattle ovaries can respond to exogenous gonadotropins. Furthermore, in most publications, the number of follicles aspirated and oocytes recovered were significantly greater than the average for adults [4,9,32,51].

Initially, short protocols in which gonadotropins were administered, starting 36–48 h before LOPU, were most popular because of their simplicity and effectiveness in generating large populations of follicles for aspiration. Some of these protocols used FSH alone (e.g., 100 mg total FSH divided into 4 injections 12 h apart), and some used a combination of FSH and eCG (400 IU). However, as previously indicated, studies in heifers and cows showed better results when oocytes were sourced from larger follicles [39,40,41]. Consistent with these reports, our work with Holstein calves of 2–6 months of age showed higher rates of development to the blastocyst stage when oocytes came from calves receiving longer gonadotropin stimulation (3 days) compared with short (2 days) or non-stimulated, which was associated with a higher proportion of larger follicles [15]. Moreover, embryo development was significantly higher with oocytes recovered from large (> 5 mm) follicles (21%) compared with those from small follicles (11%, *p* < 0.05) [5].

## 3. Laparoscopic Ovum Pick-Up

**Figure 1:**
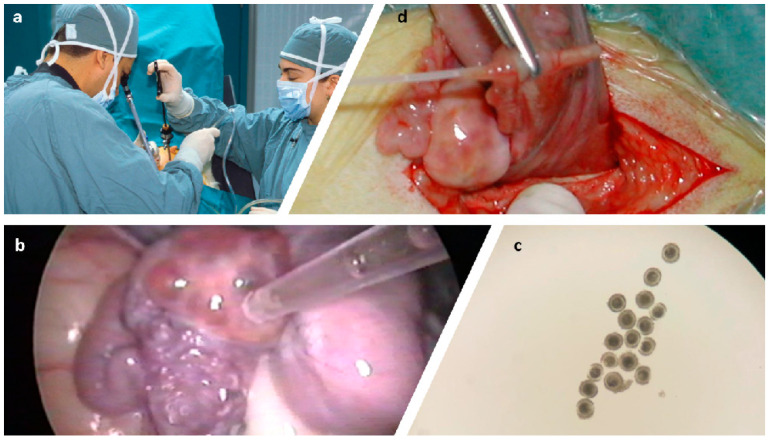
Laparoscopic ovum pick-up (LOPU) in goats: (**a**) three ports are inserted into the goat—one for the laparoscope, one for the grasping forceps, and one for the aspirating pipette/needle; (**b**) endoscopic view showing the column of follicular fluid aspirated into the pipette as the needle perforates the follicular wall; (**c**) oocytes recovered with moderate preservation of cumulus cells; and (**d**) oviduct transfer of early staged embryos.

**Figure 2:**
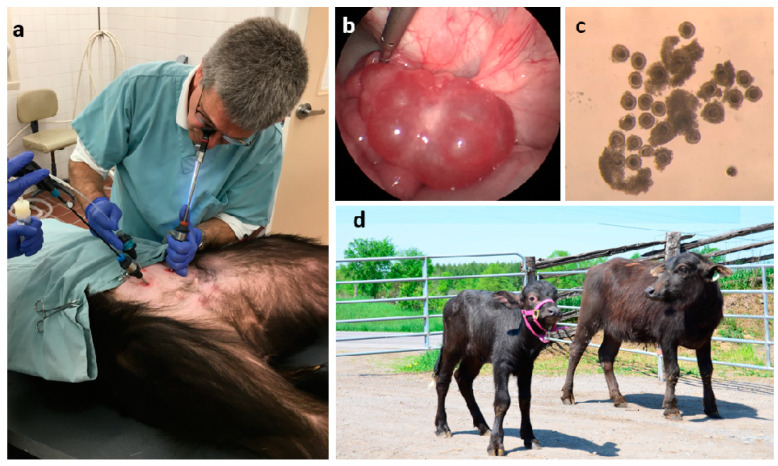
LOPU in buffalo calf: (**a**) the procedure showing port insertion and all instruments in place; (**b**) endoscopic view of hormonally stimulated ovary ready for LOPU with multiple follicles of >5 mm; (**c**) cumulus–oocyte complex recovered from buffalo calf; (**d**) “Jeanette” (left), the first buffalo born in North America by applying LOPU-in vitro embryo production (IVEP) in a prepubertal donor is pictured here with her genetic mom “Quiche” (right), who was only 4 months of age when she donated the oocytes.

The procedure is essentially the same for all species and ages described herein. To facilitate ovarian visualization and prevent complications during anesthesia (e.g., regurgitation), animals must be deprived from hay, grain, and water for at least 36, 24, and 12 h, respectively. For very young animals that are still on milk or milk replacer, they can have a last meal 12 h prior to surgery. LOPU must be conducted under general anesthesia and different anesthesia protocols may be used. In our most current protocol, we induced anesthesia to allow intubation with a mixture composed of 2 mg/KBW ketamine, 0.1mg/KBW diazepam, and 0.05 mg/KBW xylazine administered intravenously, and we maintain anesthesia with 2% isoflurane. Once under, the animals are restrained on a cradled table in Trendelenburg position and the ventral area cranial to the udder is shaved and disinfected with 2% chlorhexidine followed by 1% iodine solution. Under laparoscopic observation, all follicles of ≥2 mm in diameter are aspirated using a 20G needle mounted on an acrylic pipette connected to a collection tube and a vacuum pump (Figure 1 and Figure 2). The laparoscopic equipment consists of a 5 mm/0° laparoscope, three trocar/cannula ports, an atraumatic grasping forceps, and a cabled light source. The grasping forceps is used for pulling the mesosalpinx in different directions to expose the different surfaces of the ovaries, thereby allowing the aspiration of all follicles ≥2 mm. In our pump (WTA, Brazil), the vacuum pressure is adjusted to 50–70 mmHg, however, this may vary significantly between the pumps and tubing used. We believe the universal value should be flow and what we use is a flow rate of 60 drops of media reaching the collection tube per minute, which is approximately 3 mL/min. We insert a flow valve into the vacuum tubing between the pump and the collection tube to manage flow regulation. There are different options for the oocyte aspiration medium. We use Hepes-buffered TCM 199 or Thyrode’s-lactate (TALP) supplemented with 10 U/mL heparin, 25 μg/mL gentamicin, and 0.1% polyvinyl alcohol or bovine serum albumin (BSA). It is recommended to rinse the pipette/tubing by aspiration of medium every 10 follicles and/or at the time of switching between ovaries. After all follicles are aspirated, the ovarian surface is rinsed with warm saline solution using a pipette introduced through one of the cannula ports. Once the procedure is completed, all instruments are removed, trocar incisions are closed with a suture stitch or surgical glue, and animals are administered a preventative dose of antibiotic (e.g., 20 mg/KBW long acting oxytetracycline) and analgesic (e.g., 1 mL/45 KBW flunixin meglumine) subcutaneously.

The procedure is minimally invasive and has been shown to be very safe, specifically with no intraoperative complications nor sequels with the potential for impact on the reproductive future of the animals. In goats, it has been repeated ~10 times in the same year without issues nor a decrease in response and recovery rate [52]. Similarly, no sequels of concern were observed in sheep subjected to repeated LOPU [10]. In prepubertal cattle and buffalo, we conducted between 6 and 9 LOPU procedures/animal in a 3–4 month period, and none of them had problems producing more embryos by OPU and/or getting pregnant later in their life [5,15]. We believe the success in preservation of fertility and absence of surgical sequels is associated with the fact that the ovarian stroma is never perturbed during the procedure because the needle only penetrates the follicular wall, thereby resulting in very little to no bleeding. In addition, rinsing the ovarian surface with saline after all follicles were aspirated provides an additional layer of safety for avoiding adhesions. Notably, none of these two safety elements are possible when adult animals are aspirated by conventional US-guided OPU, making it more prone to adhesions.

## 4. In Vitro Embryo Production

Following LOPU, oocytes are subjected to in vitro maturation, fertilization, and culture prior to transferring the resulting embryos into recipients.

### 4.1. Sheep and Goats

In vitro maturation (IVM) is regularly performed in 50 µL drops of maturation medium under mineral oil. The maturation medium consists of M199 supplemented with LH (0.02 U/mL), FSH (0.02 U/mL), 17β-estradiol (1 µg/mL), sodium pyruvate (0.2 mM), cysteamine (100 µM), gentamycin (50 µg/mL), and 10% heat-inactivated fetal bovine serum. For goats, when possible, the serum is replaced with 10% heat-inactivated estrus goat serum, which promotes better nuclear maturation and acquisition of competence [8]. In vitro maturation is performed at 38.5°C in humidified atmosphere with 5% CO_2_ in air for 24 h. In vitro fertilization (IVF) is conducted in TALP medium in goats [8], and in mSOF medium in sheep [53], in both cases supplemented with 2–20% estrus goat/sheep serum. IVF takes place in 50 µL drops of fertilization medium under mineral oil at 38.5 °C in a humidified atmosphere with 5% CO_2_ in air for 15–20 h. In both species, but perhaps more critically in the goat, IVF is the bottleneck of the in vitro embryo production process. Three elements play a very important role in determining the outcome of an IVF session: semen source and type (e.g., fresh vs. frozen); estrus serum source and concentration; and, if required, the use of chemicals for capacitation (e.g., heparin). Finding the right balance between these three components of IVF will allow avoiding low fertilization rates and/or high polyspermy rates, which are the two main sources of failure. Nonetheless, there is no universal recipe, and the right protocol must be found for each male/semen batch before the results can be optimized (>60% cleavage rate). In both species, in vitro culture (IVC) is conducted in drops of IVC medium under mineral oil at 38.5 °C in a humidified atmosphere incubator with 5% O_2_, 5% CO_2_, and 90% N_2_. The IVC medium is mSOF medium, which is the original medium reported by Tervit et al. [53], supplemented with 2% essential (BME) and 1% non-essential (MEM) aminoacids and 8 mg/mL fatty acid free BSA. In some protocols, BSA is replaced with 5% fetal bovine serum on day 5 of culture to promote final development to blastocyst. With good laboratory practices, cleavage rates of >60% and blastocyst rates of >25% are consistently achieved in sheep and goats [3,11,19,27,28,46,49,54,55,56].

### 4.2. Prepubertal Cattle and Buffalo

In our work with prepubertal dairy cattle and buffalo, we used standard commercial bovine media and procedures with minor deviations from those previously published [57]. This is consistent with publications by others, who also showed little to no deviation in the media and procedures used for IVM/F/C of calf oocytes compared with those in use for adult cows and mature heifers [26,30,58,59,60,61]. One deviation was the addition of antioxidants to the maturation medium in buffalos, which has been shown to be of great importance given the high lipid content of buffalo oocytes, making them more susceptible to peroxidation damage [62]. The second deviation was to reduce the number of sperm during IVF of Holstein calf oocytes to help control the incidence of polyspermy.

During our studies, we fixed and stained a subset of calf oocytes following IVM/IVF for the purpose of learning more about how they performed and what issues were we experiencing. In the case of Holstein calves, we learned that ~80% of the oocytes were able to complete nuclear maturation. This suggests that this aspect of oocyte competence, i.e., the ability to resume meiosis and re-arrest at metaphase II with extrusion of the first polar body, may be adequate in calf-oocytes. However, following IVF, we found a high incidence of polyspermy with a range of 20–45%, suggesting calf oocytes are not fully competent to undergo the cortical reaction following fertilization. This finding is consistent with electron microscopy studies showing cortical granules are present in a lower number and poorly distributed in calf oocytes by comparison with those of adults [31]. Interestingly, higher rates of polyspermy were unaffected by the hormonal stimulation regime, but normal fertilization increased as the animals grew older, and could be improved by lowering the insemination dose at IVF [5,15].

Overall, in Holstein calves, we obtained cleavage rates of 60–70% and blastocyst rates around 20%. The hormonal stimulation protocol had an influence on embryo yields, with longer protocols and protocols combining FSH and eCG resulting in higher blastocyst rates compared with shorter protocols and FSH alone. This is consistent with longer protocols resulting in a higher proportion of larger follicles, which has been associated with a higher proportion of oocytes capable of development in prepubertal [15,41] and in older animals [40,63].

In the case of oocytes from Mediterranean buffalo calves, only 50% of the oocytes were capable of nuclear maturation, indicating that further optimization of stimulation protocols and IVM conditions is necessary for increased IVEP efficiency. Moreover, polyspermy rates (10–45%) were similar to those described above for Holstein calves and subject to the same variables (e.g., age and semen dose), and blastocyst yield was lower, around 10%.

## 5. Embryo Transfer

### 5.1. Sheep and Goat

In small ruminants, embryo transfer into recipients requires surgery under general anesthesia, and it is a matter of debate to transfer early embryos into the oviduct, or more developed embryos into the uterus. Those who advocate for a longer culture period will argue that it allows selecting the best suited embryos for transfer, thereby needing fewer recipients. Moreover, if the interest is in the production of frozen/vitrified embryos for transfer at a different time or location, then longer culture is required because good cryopreservation rates are only achieved with compact morula/blastocyst-staged embryos. However, in large commercial programs with the number of offspring born as the main success indicator, we believe that more offspring are born when “Mother Nature” is used as an incubator. Early embryo transfer is normally conducted on day 2 (day 0 = IVF) when most of the cleaved embryos are in the four-cell stage. At this stage, embryo quality/viability is rather difficult to assess, so it is standard to transfer 3–4 embryos per recipient as, most of the time, only ≤50% of the embryos implanted will develop to term. Embryo transfer at this stage requires implantation into the oviduct of a synchronized recipient. For that matter, under general anesthesia and by means of a mid-ventral laparotomy, the reproductive tract is exteriorized, and a Tomcat^®^ (Sherwood, St. Louis, MO, USA) loaded with the embryos is threaded into the oviduct through the fimbria to allow transferring the embryos (Figure 1d). For late embryo transfer, the embryos are cultured for 6 days to allow further development and those that reached compact morula/blastocyst stages are transferred into the uterus of a synchronized recipient. For that purpose, under laparoscopic observation and using a grasping forceps, the tubal end of the uterine horn is exteriorized through a small incision (2 cm), then the horn is perforated with an 18G blunt needle to allow a Tomcat^®^ catheter with the embryos to be introduced through that hole, followed by discharging the embryos into the uterine lumen. In both cases, prior to embryo transfer, the recipients are explored by laparoscopy to confirm the presence of at least one morphologically sound corpus luteum, and only the horn/oviduct ipsilateral to the CL is exteriorized. Overall, pregnancy rates range from 50 to 80% [3,19,28] for embryos transferred fresh and 40 to 50% for frozen/vitrified embryos [64,65,66,67,68].

### 5.2. Prepubertal Cattle and Buffalo

One of the most interesting findings from our studies is the fact that, even if the proportion of competent oocytes collected from calves is lower than in adults, the embryos produced from developmentally competent oocytes are equally capable of full term development as those of adult cows. Initially, we transferred 21 Holstein calf-derived blastocysts into an equal number of recipients, of which 13 became pregnant (62%) and all were able to go to full term and delivered healthy calves [15]. More recently, we transferred >100 calf-derived embryos and pregnancy rates have been ~50% (Baldassarre et al., unpublished data). In the case of Mediterranean buffalo, we had very limited access to adult recipient animals in the past, but 3 of 10 recipients transferred became pregnant (30%) and all delivered healthy calves.

## 6. Balance and Perspectives for the Future of the Technology

### 6.1. Small Ruminants

LOPU followed by IVEP has the potential to produce more offspring from superior sheep and goats than conventional MOET, mainly because it can be repeated more times and more often in the reproductive life of the donors. It also allows the production of progeny from elite animals that are not eligible for MOET such as prepubertal, senile, and donors that have repeatedly failed to produce transferable embryos by MOET. Yet, the technology is far from been widely used in commercial programs aimed at accelerating genetic gain, which is a significant contrast from what happens in the cattle industry. According to the embryo production statistics of the International Embryo Transfer Society [69], in vitro embryo production worldwide has grown exponentially in the last decade to the point where, starting in 2016, the number of in vitro produced embryos has exceeded the number of conventional in vivo produced embryos, and has stayed like that since then. When comparing the results/efficiencies obtained in small ruminants with those of cattle, one can argue that there is no technical or scientific limitation to justify the lack of commercial development for this technology. A more in-depth analysis may point at an insufficient number of veterinarians trained to conduct LOPU, the lack of laboratories specialized in small ruminant offering IVEP services for practitioners, and the relative value of animals that may not justify the extra costs of implementing this technology.

### 6.2. Prepubertal Cattle and Buffalo

The technology has reached an efficiency point that justifies its implementation in commercial applications, as reflected by some of the largest genetic companies having ongoing programs for accelerated gain based on calf-LOPU. In the Holstein breed, if calves are collected by LOPU every 2 weeks between 2 and 6 months of age (total of 8 LOPU), at the current level of efficiency, the expectation would be to obtain on average a total of 180 oocytes/calf and following IVM/F/C at least 25 transferable blastocysts/calf, which would result in at least 12 calves born following transfer to recipients. These calves would be born before or around the time that the mother reaches the age and weight to be bred to the first time, which is the true power of this technology, i.e., the ability to reach the market faster with the new generation of elite genetics. In the case of Mediterranean buffalo, the technology needs further refinement in order to improve the embryo yields, but even at the current stage of efficiency, also between 2 and 6 months of age, one would expect to collect on average >100 oocytes and convert that into at least five calves born before their genetic mothers are ready for first breeding.

We believe there are at least two major areas with potential for improvement of results through further research. A common goal is the improvement in the proportion of oocytes that are competent for full development, which is more critical in the case of prepubertal animals. One area is through hormonal priming, i.e., better conditioning of the ovaries to stimulate increased intrafollicular acquisition of oocyte developmental competence. As mentioned earlier, one target is increasing the proportion (and size) of large follicles, as there are no doubts this is linked to oocyte competence. This could be achieved by means of modifying/extending the period of gonadotropin stimulation. The second area with potential for further improvement is the development of IVM protocols tailored for promoting acquisition of competence, especially for oocytes collected from prepubertal donors. These should include strategies for delaying nuclear maturation and improving cytoplasmic maturation, as well as supplementation of the IVM medium with growth factors, cytokines, and embryokines that have the potential for allowing increased accumulation of critical molecules associated with the acquisition of competence in the ooplasms.

## 7. Conclusions

The LOPU-IVEP technology is an efficient tool for accelerated genetic gain in ruminant reproductive programs. In small ruminants, it has been technologically developed to a commercial level with embryo yields and pregnancy rates like those obtained in cattle. However, for logistical and cost-related reasons, the industry has been deprived from a widespread commercial application of the technology. On the prepubertal bovine side, the technology has reached commercial levels of efficiency and has substantial potential for doubling or even tripling its performance in the next few years. Some of the research may continue to occur at an academic level, but it is the large genetic corporations that need to decide if it is worth having those elite “calves from calves” born earlier, thereby justifying the financial commitment for advancing the research.

## Data Availability

No new data were created or analyzed in this study. Data sharing is not applicable to this article.
